# Psychotropic medication treatment patterns in community-dwelling schizophrenia in China: comparisons between rural and urban areas

**DOI:** 10.1186/s12888-019-2217-1

**Published:** 2019-08-05

**Authors:** Cai-Lan Hou, Shi-Bin Wang, Fei Wang, Ming-Zhi Xu, Miao-Yang Chen, Mei-Ying Cai, Yao-Nan Xiao, Fu-Jun Jia

**Affiliations:** 1 Guangdong Mental Health Center, Guangdong General Hospital & Guangdong Academy of Medical Sciences, 7/F, No.123, Huifuxi Road, Guangzhou, Guangdong Province China; 2 The Third People’s Hospital of Luoding, Luoding, Guangdong Province China; 3 Guangzhou Yuexiu Center for Disease Control and Prevention, Guangzhou, Guangdong Province China

**Keywords:** Community-dwelling, Schizophrenia, Prescription, Rural, Urban

## Abstract

**Background:**

To date no study has compared more specifically the psychotropic medication treatment patterns for patients with schizophrenia living in community between rural and urban areas. This study examined the rural-urban differences of the use of psychotropic drugs among community-dwelling individuals with schizophrenia in China.

**Method:**

Data on 993 community-dwelling patients with schizophrenia (*n* = 479 in rural area and *n* = 514 urban area) were collected by interviews during 2013–2014, and 2015–2016 according to the diagnosis of DSM-IV or ICD-10. Data on patients’ socio-demographic and clinical characteristics, prescriptions of psychotropic drugs were collected using a standardized protocol and data acquisition procedure.

**Results:**

Multivariate analyses revealed that in comparison with the rural counterparts, the patients from the urban area were significantly more frequently prescribed antipsychotic polypharmacy, clozapine, and benzodiazepines, but the patients from the rural area had more frequently prescribed anticholinergics.

**Conclusions:**

Substantial variations in psychotropic medication treatment patterns for patients with schizophrenia living in community were found between rural and urban areas in China. Common use of antipsychotic polypharmacy, clozapine and benzodiazepines in urban area, and anticholinergics in rural area need to be further addressed.

## Background

There exist significant variations in the socio-economic status, culture, demographics and history of urban and rural places which influence mental health, while access to appropriate health services and health professionals are important determinants of treatment of schizophrenia.

Most of the studies on urban-rural differences in patients with schizophrenia focused on their epidemiological and aetiological factors [1–3], but urban-rural differences in psychotropic drug treatment model for people with schizophrenia living in the community are not well studied.

A number of factors associated with the availability, organization, and delivery of mental health services in rural and urban communities could also explain the differences in psychotropic use [3]. Rural communities may encounter place-specific challenges to population health, including geographic isolation, or stagnant economic growth in some areas [4]. Especially in China, general practitioners (GPs) have no permission of prescribing anti-psychotic medications except for benzodiazepines. Fewer psychiatrists in rural area than in urban area may also affect the medication treatment pattern.

It is attractive to explore differences in psychotropic medication treatment patterns and investigate related factors that may be the basis for any differences observed. Therefore, in the present study, we addressed two questions including what was the medication treatments currently used in community settings in urban and rural areas respectively and what factors were associated with choice of medication strategy in urban and rural area?

## Methods

### Research design and subjects

This study was an extension of a cross-sectional pharmaco-epidemiological survey on prescription trends of psychotropic medications [5] conducted between August, 2013 and March 2016 in Guang Zhou and Luo Ding, Guangdong province, China. Guang Zhou is the capital city of Guangdong province that is an urban area, while Luo Ding, a typical rural area lies in western Guangdong province and is underdeveloped.

Patients who met the following criteria were consecutively recruited: (1) aged equal to or greater than 18 years; (2) being diagnosed as schizophrenia according to DSM-IV or ICD-10; (3) being treated by primary care physicians; (4) having ability to understand the content of the interview and (5) being able to provide written informed consent. If the patients were antipsychotic-free, they would be excluded from the study. The study protocol was approved by the Ethics Committees of Guangdong Mental Health Centre (ethical code is Z2013–002). All patients provided written informed consent.

In China, most of the community-dwelling patients with schizophrenia who have presented to hospitals and primary care services are registered in the Chinese National Psychiatric Management System. The recruitment processes of this cross-sectional study were as follows: 22 primary care service centers in Guangzhou (urban area) and 21 primary care service centers in Luo Ding (rural area) were selected randomly. Primary care physicians in the selected service centers contacted the randomly selected patients by telephone to provide a detailed introduction about this study procedure. Six training psychiatrists conducted interviews with the patients. The initial sample size is 1200, and 600 patients in urban and rural areas, respectively.

### Assessments

Basic socio-demographic and clinical data including prescription of psychotropic drugs were collected by a review of their electronic medical records. We only recorded and analyzed the present prescribing condition (active medications). Doses of antipsychotic drugs were converted into the prescribed daily dose/the defined daily dose ratio (PDD/DDD ratio) [5–7].

This study focused on the following major groups or aspects of treatments viz. second generation antipsychotics (SGAs) except clozapine, antipsychotic polypharmacy, clozapine, first generation antipsychotics (FGAs), antipsychotics free, benzodiazepines, mood stabilizers, antidepressants and anticholinergics. Clozapine was singled out due to its special place in the use of treatment-resistant patients. Antipsychotic polypharmacy (APP) was defined as co-prescription of 2 or more antipsychotics [5, 8].

The Brief Psychiatric Rating Scale (BPRS) was administered to assess psychotic symptoms [9, 10]. Adverse effects of antipsychotics were assessed using the Simpson and Angus Scale of Extrapyramidal Symptoms (SAS) [11]. The 10-item Montgomery-Asberg Scale (MADRS) was used to measure depressive symptoms [12, 13].

### Statistical analysis

SPSS 20.0 for Windows was used to analyze the data. The comparisons between rural and urban patients with regard to basic socio-demographic and clinical characteristics were performed using independent sample t-test, U test, and chi-square test, as appropriate. Multiple logistic regression analyses with the “Enter” method was conducted to examine the independent relationships between areas and prescribing patterns. For antipsychotic polypharmacy, participants were classified as APP and not APP categories. SGA and FGA were also classified as binary variables. Any use of SGAs except clozapine, antipsychotic polypharmacy, clozapine, FGAs, antipsychotic free, benzodiazepine, mood stabilizers, antidepressants and anticholinergics were entered as the dependent variables separately, while rural/urban area was an independent variable; demographic variables and clinical characteristics that showed significant differences between the two groups in aforementioned univariate analyses were adjusted for as covariates. The two-tailed significance level was set at 0.05.

## Results

In the end, a total of 993 patients with schizophrenia (514 in urban and 479 in rural areas) were available and included in the analyses. The response rate was 85.67% in urban areas and 79.83% in rural areas, respectively.

Socio-demographic and clinical data of the whole sample and also separately by urban and rural areas were showed in Table 1. There were significant differences between the two groups in terms of gender, marital status, first episode, personal income, coverage of health insurance, family history of psychiatric disorders, major medical conditions, use of clozapine, SGAs (except clozapine), APP, antidepressants, benzodiazepines and anticholonergics, age, education level, illness duration, BMI, severity of depressive, negative and anxiety symptoms, and extrapyramidal symptom. Multiple logistic regression analyses revealed that after controlling for covariates, compared to rural patients, urban patients were more likely to receive APP, clozapine, and benzodiazepines, but had less frequently prescribed anticholinergics (Table 2).

**Table 1:** Socio-demographic and clinical characteristics and psychotropic drug prescription in urban and rural areas ^a^

	Total (*N* = 993)		Urban (*N* = 514)		Rural (*N* = 479)		X^2^	*df*	*P*
	n	%	n	%	n	%			
Male gender	586	59.0	273	**53.1**	313	**65.3**	15.3	1	**< 0.001**
Married	393	39.6	182	**35.4**	211	**44.1**	7.7	1	**0.005**
Employed	661	66.6	342	66.5	319	66.6	0	1	0.98
First episode	219	22.1	163	**31.7**	56	**11.7**	57.8	1	**< 0.001**
Personal income over 3000 yuan	24	2.4	19	**3.7**	5	**1.0**	7.3	1	**0.007**
No health insurance	126	12.7	122	**23.7**	4	**0.8**	117.3	1	**< 0.001**
Psychiatric family history	195	19.6	137	**26.7**	58	**12.1**	25.6	1	**< 0.001**
Major medical conditions	231	23.3	201	**39.1**	30	**6.3**	149.8	1	**< 0.001**
Clozapine	357	36.0	222	**43.2**	135	**28.2**	24.2	1	**< 0.001**
FGA	431	43.4	231	44.9	200	41.8	1.02	1	0.31
SGA except Clozapine	411	41.4	161	**31.3**	250	**52.2**	44.5	1	**< 0.001**
APP	310	31.2	193	**37.5**	117	**24.4**	19.8	1	**< 0.001**
Antidepressants	40	4.0	28	**5.4**	12	**2.5**	5.5	1	**0.01**
Mood stabilizers	189	19.0	108	21.0	81	16.9	2.7	1	0.1
Benzodiazepine	161	16.2	128	**24.9**	33	**6.9**	59.2	1	**< 0.001**
Anticholinergics	432	43.5	187	**36.4**	245	**51.1**	21.9	1	**< 0.001**
	Mean	SD	Mean	SD	Mean	SD	*t/u*	*df*	*P/Z*
Age,y	43.2	11.6	**46.8**	10.2	**39.4**	11.8	10.6	991	**< 0.001**
Education,y	9.4	2.7	**10.5**	2.8	**8.3**	2.2	13.3	991	**< 0.001**
Age of onset,y	25.6	9.1	25.1	8.9	26.0	9.3	−1.6	991	0.103
Illness duration,y	17.6	11.1	**21.7**	10.3	**13.3**	10.1	12.8	991	**< 0.001**
No. Of admissions	2.2	2.4	2.4	2.7	2.1	2.03	−1.1	...	0.26^b^
BMI,kg/m^2^	23.6	4.1	**24.7**	4.8	**22.5**	2.4	8.7	985	**< 0.001**
**PDD/DDD**	**0.9**	**1.2**	**0.9**	**1.6**	**0.8**	**0.5**	**1.7**	**991**	**0.08**
MADRS total score	7.4	8.2	**10.01**	9.3	**4.7**	5.7	10.6	991	**< 0.001**
BPRS score									
positive	5.9	2.8	6.1	3.1	5.8	2.4	1.9	991	0.053
negative	5.7	2.8	**6.2**	3.08	**5.3**	2.5	4.8	991	**< 0.001**
anxiety	4.2	2.03	**3.2**	1.7	**5.2**	1.8	−18.2	991	**< 0.001**
SAS total score	11.7	4.2	**5.2**	0.2	**2.2**	0.1	9.8	991	**< 0.001**

*Abbreviations*: *APP* antipsychotic polypharmacy, *BMI* body mass index, *BPRS* brief psychiatric rating scale, *FGA* first-generation antipsychotic, *MADRS* Montgomery-Asberg Depression Rating Scale, *PDD/DDD* prescribed daily dose/the defined daily dose ratio, *SAS* Simpson and Angus scale of extrapyramidal symptoms, *SGA* second-generation antipsychotic

^a^ Boldface indicates a statistically significant difference (*P* < 0.05)

^b^ Mann-Whitney *U* test was used

**Table 2:** The independent associations of psychotropic drug prescription between rural/urban area

	*P*	Odds ratio	95% C.I.
SGA, except clozapine ^a^			
Rural	0.92	1.02	0.67–1.55
Urban	–	1	–
Antipsychotic polypharmacy-APP ^a^			
Rural	0.002	0.5	0.32–0.77
Urban	–	1	–
Clozapine ^a^			
Rural	0.003	0.52	0.34–0.81
Urban	–	1	–
FGA ^a^			
Rural	0.08	0.71	0.47–1.05
Urban	–	1	–
Benzodiazepines ^a^			
Rural	< 0.001	0.28	0.16–0.51
Urban	–	1	–
Mood stabilizer ^a^			
Rural	0.32	0.76	0.44–1.31
Urban	–	1	–
Antidepressants ^a^			
Rural	0.15	0.44	0.14–1.34
Urban	–	1	–
Anticholinergics ^a^			
Rural	0.04	1.503	1.01–2.24
Urban	–	1	–

*Abbreviations*: *APP* antipsychotic polypharmacy, *FGA* first-generation antipsychotic, *SGA* second-generation antipsychotic

^a^: gender, marital status, first episode, personal income, coverage of health insurance, family history of psychiatric disorders, major medical conditions, age, education level, illness duration, BMI, and severity of depressive, negative and anxiety symptoms, as well as the extrapyramidal symptoms were controlled for as covariates

## Discussion

To the best of our knowledge, this was the first study that examined the urban–rural differences of psychotropic medication treatment patterns in Chinese community-dwelling patients with schizophrenia. We found that rural patients had less use of antipsychotic polypharmacy, clozapine, and benzodiazepine, and more use of anticholinergics by contrast with urban patients. This cross-sectional study of prescribing patterns in the real-world settings might reflect the practice and treatment patterns due to several reasons.

The possible reason for discrepancies in prevalence rates of antipsychotic polypharmacy among studies could be explained by differences in socio-demographic characteristics of the patients with schizophrenia, such as age, gender, educational level, income, and family support. A recent study reported that the place of residence was found to be remarkably related to polypharmacy and urban outpatients with schizophrenia had more APP by bivariate analysis, however multivariate logistic regression did not find the significant meaning [14]. Our finding on APP was similar to the previous study. The levels of antipsychotic polypharmacy were typical with community settings [15]. Rural communities have distinct socioeconomic and cultural characteristics from urban communities. Psychiatrists with lower education levels in rural areas may prefer monotherapy, while their urban counterparts may pursue a higher control rate and better continuous education could encourage them for polypharmacy [16]. On the other hand, patients with schizophrenia in urban areas with higher education could demand more health resources and accept higher spending on the antipsychotic for a higher quality of life [5]. In addition, relatively difficult access to psychological services and mental health professionals in rural places were perhaps influencing factors.

We also found that the SAS scores were significantly higher in urban group than in rural group, which could also be a result of the more antipsychotic polypharmacy in urban areas. However, the patients with schizophrenia from the urban areas had less frequently use of anticholinergics, which could relieve the iatrogenic extrapyramidal symptoms (EPS) of antipsychotic drugs. And there is little evidence supporting improved efficacy of APP, but it could increase adverse effect, mortality, cost of treatment and reduce treatment adherence. Additionally, the shortage of medical resources in rural areas may lead the rural psychiatric hospitals store less type and quantity of antipsychotics, rural clinical settings are more likely to have typical antipsychotics/FGAs, which could cause EPS. Besides reducing the side effects of antipsychotics, anticholinergic could also be used for COPD, asthma and Parkinson’s disease. Data showed the prevalence rates of related chronic diseases in rural areas were higher than those in urban areas [26]. So the general practitioners (GPs) in rural areas may prescribe the relatively cheap anticholinergic for comorbidities of patients with schizophrenia [19]. This merits more research and concerns by the psychiatrists and professionals.

About 30% of individuals with schizophrenia have treatment-resistant conditions, and clozapine is the only antipsychotic with known efficacy in treatment-refractory schizophrenia [17]. Previous survey in the United States found that treatment resistance and living in a rural area with historically high rates of clozapine prescription were the powerful predictors of clozapine prescription [18]. We found in this study that rural patients were prescribed less than urban patients although the price of clozapine is cheap and common use in China [19]. Although clozapine has a distinctive role in the treatment of schizophrenia because of its unique advantage, psychiatrists should pay more attention to its specific risks especially including agranulocytosis, metabolic syndrome, myocarditis and bowel obstruction [20]. Furthermore, the lack of medical human resource in rural areas could not meet the monitoring requirements for clozapine [21]. The potential risks, the mandatory blood monitoring as well as the insufficient knowledge on treatment of the risks may decrease the prescription of clozapine in rural areas. The US study reported that the geographic variation and that local practice model significantly influenced clozapine use [18]. However, there were also controversial studies showing that patients treated at university hospitals mainly located in the more urban areas were less likely to prescribed clozapine compared to individuals treated at non-university hospitals mainly located in less urban areas. This also reflected the region difference on drug prescription [22, 23].

Rural patients were prescribed less benzodiazepine in this study, showing the rural doctors perhaps were less likely to prescribe the benzodiazepines for schizophrenic patients, which was contrary to some studies. In a study of tranquillizer prescribing in two municipal and two rustic areas of the United Kingdom, Gabe and Williams found that rustic doctors were more likely to prescribe tranquilizers as their practice expanded compared to urban doctors [24]. Cutts and Tett found similar trends in their study of prescribing patterns in Queensland, Australia [25].

There were several strengths of the present study including the large, homogeneous and randomly selected sample, as well as using the standard conversion method of antipsychotic dosage. However, the results should be interpreted with cautiously due to several limitations of methodology. First, the causality between different variables was difficult to get because of this study’s cross-sectional survey. Second, only patients with schizophrenia during clinically stable phase living in communities were enrolled from one metropolitan city and one rural area. Therefore, the conclusions may not be universally applied to different parts of China and to other subject cohorts due to the limitation of representativeness. Third, there was no exact data on the proportion of the Chinese National Psychiatric Management System registered schizophrenic patients in the total patients with schizophrenia. Selection bias indeed existed due to the missing of the patients who did not access to the institutes. And due to unequal response rate between rural and urban areas (85.67% in urban and 79.83% in rural), selection bias may also exist in the study. Despite these limitations, our data have health policy implications. The marked use of APP, clozapine and benzodiazepine in urban areas in our study, and the marked use of anticholinergics in rural areas in our study may be warranted by evidence of better efficacy and relapse prevention and reduced side effects.

In conclusion, substantial variations in psychotropic medication treatment patterns for community-dwelling schizophrenia were found between rural and urban area in China. Common use of antipsychotic polypharmacy, clozapine, and benzodiazepine in urban areas and anticholinergic use in rural areas need to be further addressed. Our findings suggested that further research would be needed to investigate the determining factors for these differences in psychotropic use.

## Data Availability

All data generated and analysed during this study are included in this published article. Other data acquisition could contact the correspondents.
